# *In Vitro* Differentiation of Preosteoblast-Like Cells, MC3T3-E1, to Adipocytes Is Enhanced by 1,25(OH)_2_ Vitamin D_3_

**DOI:** 10.3389/fendo.2017.00128

**Published:** 2017-06-16

**Authors:** Elisha Pendleton, Nalini Chandar

**Affiliations:** ^1^Department of Biochemistry, Chicago College of Osteopathic Medicine, Midwestern University, Downers Grove, IL, United States

**Keywords:** vitamin D, osteoblasts, MC3T3, Rb1, adipogenic differentiation, transdifferentiation

## Abstract

Osteoblasts and adipocytes originate from common mesenchymal progenitor cells and are controlled by specific transcription factors. While 1,25-dihydroxyvitamin D_3_ (vitamin D) is known to be an important factor for osteoblast differentiation, there are conflicting reports regarding its effect on adipogenesis. In this study, we attempted to understand the effect of exposure of preosteoblasts (MC3T3-E1) to adipogenic media with and without vitamin D and determined the expression of adipogenic genes during this process. Our studies show that while transdifferentiation of preosteoblasts occurred on exposure to adipogenic media, the effect of vitamin D treatment was synergistic resulting in several hundred fold increase in adipocyte transcription factors C/EBPα and peroxisome proliferator-activated receptor-gamma (*P* < 0.001) along with an increase in markers of adipogenesis and accumulation of lipid droplets in cells. Vitamin D treatment was also accompanied by 100-fold to 700-fold increases in vitamin D receptor expression during the treatment period (*P* < 0.001). To determine how the effect of vitamin D might compare to other genetic manipulations that promote adipogenic differentiation, we stably knocked down retinoblastoma expression in MC3T3-E1 cells. Recent studies have suggested retinoblastoma (Rb1) tumor suppressor gene function to be critical to maintain osteoblasts function and inhibit adipocyte differentiation. We exposed MC3T3-E1 cells with reduced Rb1 expression to adipogenic media and found an increase in adipogenic differentiation when compared to cells with a full complement of Rb dosage. However, the extent of the change was not as dramatic as seen with vitamin D. These studies show that preosteoblasts are sensitive and respond to these manipulations that favor the adipocytic phenotype. While vitamin D is not known to directly affect targets in adipogenesis, our observations may have resulted from the malleability of preosteoblast genome in MC3T3-E1 cells, which allowed adipocyte specific gene expression under appropriate stimuli. Why this pathway is influenced and subverted by an anabolic bone factor such as vitamin D remains to be determined.

## Introduction

Osteoblasts and adipocytes are derived from mesenchymal stem cells, and many studies have suggested the presence of plasticity between these two cell types (1, 2). Aging and estrogen deficiency are also known to produce an increase in the bone marrow adipocity as well as decrease in the ability of mesenchymal precursor to progress toward osteogenesis (2, 3). Several studies have also suggested the occurrence of both osteoblastic and adipocytic phenotype in the same cell population (4). In fact, a preadipocyte cell line has been derived from osteoblastic cell population from mouse calvaria (5).

One of the factors that regulate adipogenesis is peroxisome proliferator-activated receptor-gamma (PPARγ), which belongs to the class of nuclear hormone receptors that are affected by specific ligands such as fatty acids as well as by cross talk with other molecules. PPARγ is both sufficient and necessary for conversion of preadipocytes into adipocytes and is a master regulator of adipogenesis (6).

Vitamin D is an important bone anabolic agent, and several bone specific genes are directly regulated by this hormone. 1,25-Dihydroxyvitamin D3, the active form of vitamin D functions through its receptor, vitamin D receptor (VDR) a ligand dependent transcription factor, belonging to the same class of nuclear receptor family of transcription factors as PPARs. The similarity between these two factors is that they mediate their action by heterodimerizing with retinoic acid receptor in the regulation of gene expression. Studies on vitamin D effects on osteoblasts are mixed. While some studies show vitamin D to inhibit adipocyte differentiation, other studies have suggested adipocytic conversion of bone marrow osteoblasts by vitamin D (6–8). While vitamin D has been shown to inhibit adipocytic differentiation of preadipocytes, there are studies that demonstrate a stimulatory effect on other mesenchymal derived cells (7, 8).

There is also cross talk between PPARγ and VDR as VDR elements have been discovered in the PPAR genes demonstrating an influence of vitamin D over PPAR system (9). PPARγ also appears to bind to VDR and inhibit vitamin D dependent transactivation (10).

Retinoblastoma tumor suppressor gene (Rb1) function is important for controlling the cell cycle progression and differentiation of several cell types (11). Loss of Rb1 gene function predisposes individuals to retinoblastoma and osteosarcomas which are commonly seen as secondary tumors in the pediatric population. Recent animal studies have suggested a role for pRb in the regulation of mesenchymal cell fate towards osteoblasts, by potentiating the effect of Runx2 a master regulator for osteoblast differentiation (12). pRb1 is also known to be important for adipocytic differentiation as it is required for activating several of the transcription factors involved in adipocyte differentiation (13). However, complete loss of Rb1 expression from mesenchymal stem cells predisposes them to an adipocytic cell fate (14). While the role is complex and yet to be clearly elucidated, Rb1 is generally considered to regulate cell fate and in the absence of Rb1 cells default to an adipogenic phenotype (14, 15).

In this study, we attempted to determine the effect exposure to adipogenesis promoting (AP) media on MC3T3-E1 preosteoblasts. We also determined how the presence of vitamin D might affect the expression of adipocytic phenotype. The effect of Rb1 deficiency in osteoblasts and their transdifferentiation ability to adipocytes was also studied.

## Materials and Methods

### Cell Culture

The MC3T3-E1 subclone 4 (ATCC CRL-2593) is a preosteoblast cell line isolated from mouse calvaria and is a well-established model to study osteoblast differentiation. Cell lines were grown in alpha-minimum essential media (αMEM) (Life Technologies) with 10% fetal bovine serum (FBS) (Atlanta Pharmaceuticals) under 5% CO_2_ at 37°C.

### Stable Rb1 Line

MC3T3-E1 cells were used to create Rb1 deficient line using shRNA technology. A stable clone of cells containing less than 40% of wild-type Rb1 expression was used for these studies. A control MC3T3-E1 line containing a stably expressing scrambled shRNA control was also used.

### Adipogenic Media and Vitamin D Treatment

The AP media used consisted of 2% FBS and αMEM containing 0.5 mM 3-isobutyl-1-methylxanthine (Sigma-Aldrich, St. Louis, MO, USA), 1μM dexamethasone (Sigma), 10 μg/ml Insulin (Sigma). The AP media were changed every 48 h after the initial treatment, and the cells were maintained in an incubator at 37°C and 5% carbon dioxide.

Vitamin D (1,25-dihydroxyvitamin D3) was purchased from Sigma-Aldrich, St. Louis, MO, USA and utilized at a final concentration of 20 nM dissolved in ethanol in AP media. All experiments were carried out in triplicates and repeated thrice.

To analyze expression of adipocyte markers after exposure to AP media, we plated equal numbers of cells and grew them to 70% confluency in regular media (10% FBS in DMEM) and switched them to serum free for 48 h before exposing them to AP media described above. Media were changed every 48 h, and a plate was harvested each day for 6 days. A similar protocol was followed for vitamin D treatment. Control cells received the same volume of the solvent used for vitamin D (ethanol).

For analyses of adipogenic expression in Rb1 knockdown cells, we used shRNA technology to stably reduce Rb1 expression. Several clones were first characterized and analyzed, and in this study, we used a clone with a 50% stable reduction of Rb1. Control MC3T3-E1 cells carried a stable scrambled shRNA sequence. These cells were treated as described above, except the vitamin D treatment was not performed on these cells. Validation of knockdown was carried out at the level of RNA and protein.

### SyberGreen Real-time PCR

RNA was isolated using TRI reagent (Sigma). A Two-step RT-PCR was performed using Bullseye EvaGreen qPCR master Mix (Midsci) and RT-PCR Reagents Kit with Step One Plus Real Time Machine (Applied Biosystems, Roche, IN). RNA sample were converted into cDNA, using a high-capacity cDNA RT kit. Analysis of the results was performed with the 7300 RT-PCR System RQ Software version 1.4 (Applied Biosystems). Efficiency of the control and treatment samples were checked using the slope of the graph of ΔCT values versus log of the total RNA sample. If the calculated slope was less than 0.1, then comparative CT method was used to compare the gene expression between control and treatment cells. These were normalized according to a housekeeping gene, β-actin, serving as an internal control.

### Primer Pairs Used in Real-time PCR

Primers where designed using Integrated DNA Technologies, Coralville, IA, USA PrimerQuest Tool. The Genbank number was used to import the sequence, and the parameters of *T*_m_ of 60° and amplicon size of 75–125 bp were used. Sequence sets that resulted with amplicon ~100 bp were chosen. Primer pair sequences used are shown in Table 1.

**Table 1:** Mouse specific primer sequences used for quantitative real-time PCR analysis.

Primer	Sequence
VDR forward	GGCTTCCACTTCAACGCTATG
VDR reverse	TGCTCCGCCTGAAGAAACC
FABP4 forward	CCGCAGACGACAGGAAGGT
FABP4 reverse	AGGGCCCCGCCATCT
Srebf1 forward	GCGGTTGGCACAGAGCTT
Srebf1 reverse	CTGTGGCCTCATGTAGGAATACC
AdipoQ forward	GACACCAAAAGGGCTCAGGAT
AdipoQ reverse	TGGGCAGGATTAAGAGGAACA
Ucp1 forward	ACTGCCACACCTCCAGTCATT
Ucp1 reverse	CTTTGCCTCACTCAGGATTGG
C/EBPα forward	CAAGAACAGCAACGAGTACCG
C/EBPα reverse	GTCACTGGTCAACTCCAGCAC
C/EBPβ forward	GGGTTGTTGATGTTTTTGGTTT
C/EBPβ reverse	GAAACGGAAAAGGTTCTCAAAA
C/EBPδ forward	CGACTTCAGCGCCTACATTGA
C/EBPδ reverse	GAAGAGGTCGGCGAAGAGTT
Peroxisome proliferator-activated receptor-gamma (PPARγ) forward	TGAAAGAAGCGGTGAACCACTG
PPARγ reverse	TGGCATCTCTGTGTCAACCATG
β-Actin forward	TGTCCACCTTCCAGCAGATGT
β-Actin reverse	AGCTCAGTA ACAGTCCGCCTAG

*Primer sequences were designed using PrimerQuest tool (Integrated DNA Technologies) as described in Section “Materials and Methods*”.

### Protein Isolation and Western Blot Assay

Cells were lysed with buffer, containing 50 mM DTT, 2% SDS, 10% glycerol, 65 mM Tris–HCl pH 6.8, with protease inhibitor cocktail (cOmplete ULTRA Tablets Mini Roche protease inhibitor). The lysate was collected and sonicated with a Bioruptor 300 (Diagenode, NJ, USA). Protein concentrations were measured, and a standard curve was generated using a Pierce BCA Protein Assay Kit (Thermo Scientific). Rabbit VDR (Proteintech, Chicago, IL, USA) and Rabbit C/EBPα (Proteintech) were used. SuperSignal^®^ West Pico Chemiluminescent Substrate (Thermo Scientific) was used to visualize the membrane using BioRad ChemiDoc MP Imaging System (BIORAD, CA, USA).

### Luciferase Assay

Luciferase assay for PPARγ activity was conducted with PPARγ luciferase construct (a kind gift of Dr. B. Spiegelman, Addgene #1015) using BrightGlow (Promega, WI, USA) and measured with a GLOMAX 20/20 Luminometer. Transfections were carried out on MC3T3 E1 cells (control) or cells with stable Rb1 knockdown using Superfect (Qiagen) according to manufacturer recommendations. Forty-eight hours after transfection, cells were trypsinized, pelleted, and then resuspended in serum free media. An aliquot was used to count cells for normalization, the rest was assayed using Promega’s Bright-GloTM Luciferase Assay Kit (Promega, cat. no. E2610). Equal volumes of the resuspended cells and Bright-GloTM were mixed and incubated for 2 min. Samples were read for 10 s, each after a 2 s delay. Luciferase readings for each of the samples were then normalized according to the number of cells per milliliters. All measurements were carried out on triplicate samples, and experiments were repeated at least thrice.

### Statistical Analyses

Data were analyzed by one or two way ANOVA for effects of vitamin D, media type (regular media, adipogenic media) and/or time point (as appropriate) using GraphPad Prism 7 statistical software (GraphPad Software Inc., La Jolla, CA, USA) A *post hoc* Bonferroni’s test was used when appropriate. Data are expressed as Mean ± SE. A *P* value of <0.05 was considered significant.

## Results

**Figure 1:**
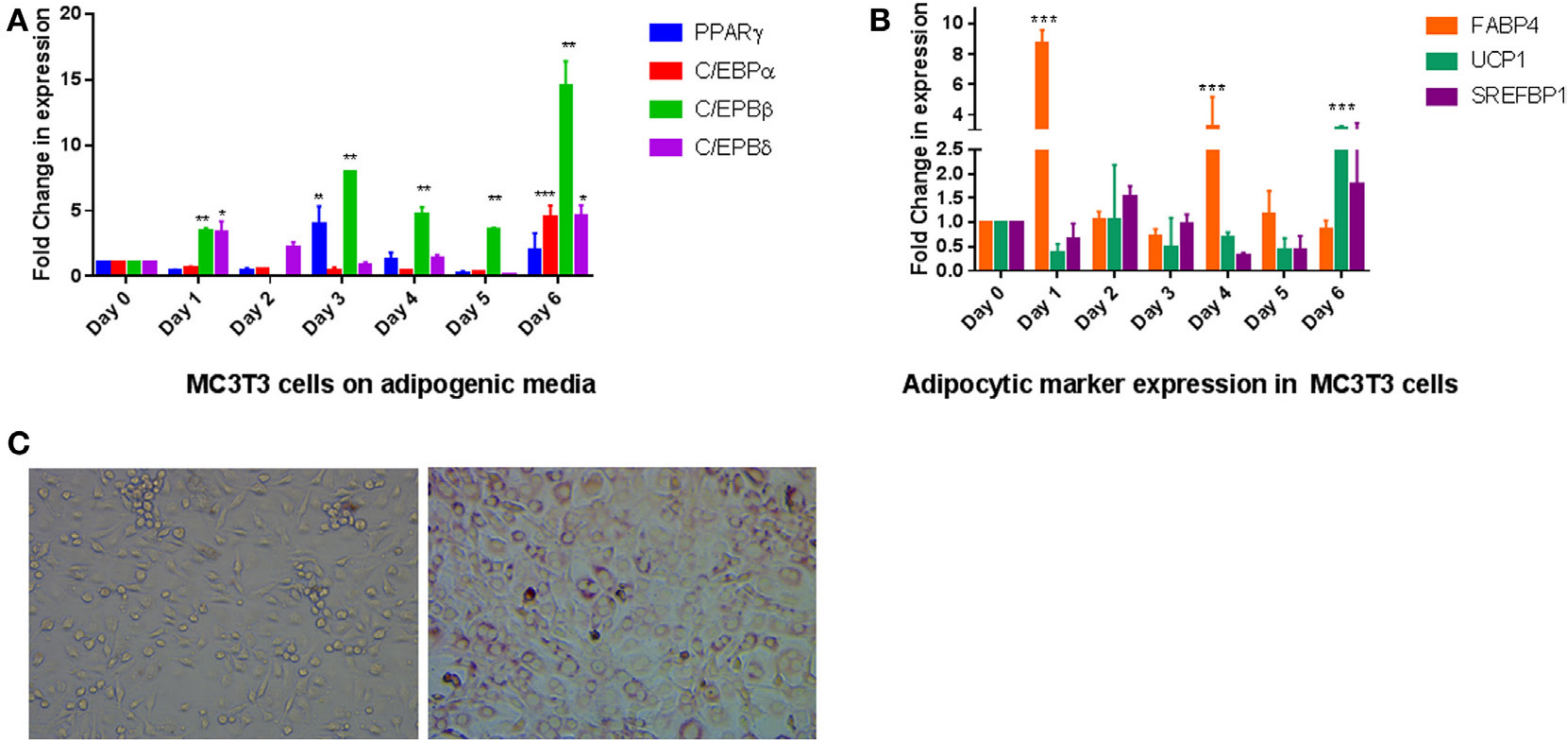
Effect of adipogenesis promoting (AP) media on MC3T3-E1 cells: **(A)** quantitative real-time PCR of key transcription factors during exposure of MC3T3-E1 cells AP media. Treatment was carried out for a week with regular changes in media, and the mRNA levels were determined. Relative mRNA expression was normalized to control β-actin, and fold inductions were calculated in reference to 0 day control which is set as 1. Results are expressed as mean ± SE (*n* = 3), ***P < 0.001, **P < 0.01, *P < 0.05. **(B)** Real-time PCR of adipocyte markers after exposure to AP media. Other details as shown in panel **(A)**. **(C)** Oil Red O staining of MC3T3-E1 cells show differentiation to adipocytes. Cells on the left were exposed to regular media for 6 days while cells on the right were exposed to AP media for the same amount of time followed by staining with ORO as described in Section “Materials and Methods” (20× magnification).

To elucidate if exposure of AP media to the preosteoblast cell line MC3T3-E1 produced transdifferentiation to an adipocytic phenotype, we exposed cells for 6 days to the media and analyzed RNA expression for several transcription factors that regulate adipogenic differentiation. The transcriptional program regulating adipogenesis is well understood and proceeds through sequential expression of different transcription factors (16). Our data on MC3T3- E1 cells followed the expected changes that occur during adipogenesis where C/EBPβ and C/EBPδ expression precedes expression of PPARγ and C/EBPα. As seen in Figure 1A, the two transcription factors (C/EBPβ and C/EBPδ) were increased within a day of treatment to about fourfold and a steady increase that continued through the treatment (*P* < 0.01 for C/EBPβ, *P* < 0.05 for C/EBPδ). CEPBβ showed a larger increase (15-fold) than C/EBPδ (5-fold). This was followed by an increase in PPARγ after 3 and 6 days of treatment (*P* < 0.01). An increase in C/EBPα was observed on Day 6 on AP media. The changes in these transcription factors were accompanied by an increase in expression of several adipocyte markers such as FABP4, UCP1, and SREFBP1 (Figure 1B). Positive Oil Red O staining confirmed the presence of lipid droplets, which were observed in MC3T3-E1 cells exposed to AP media but not regular media (Figure 1C).

**Figure 2:**
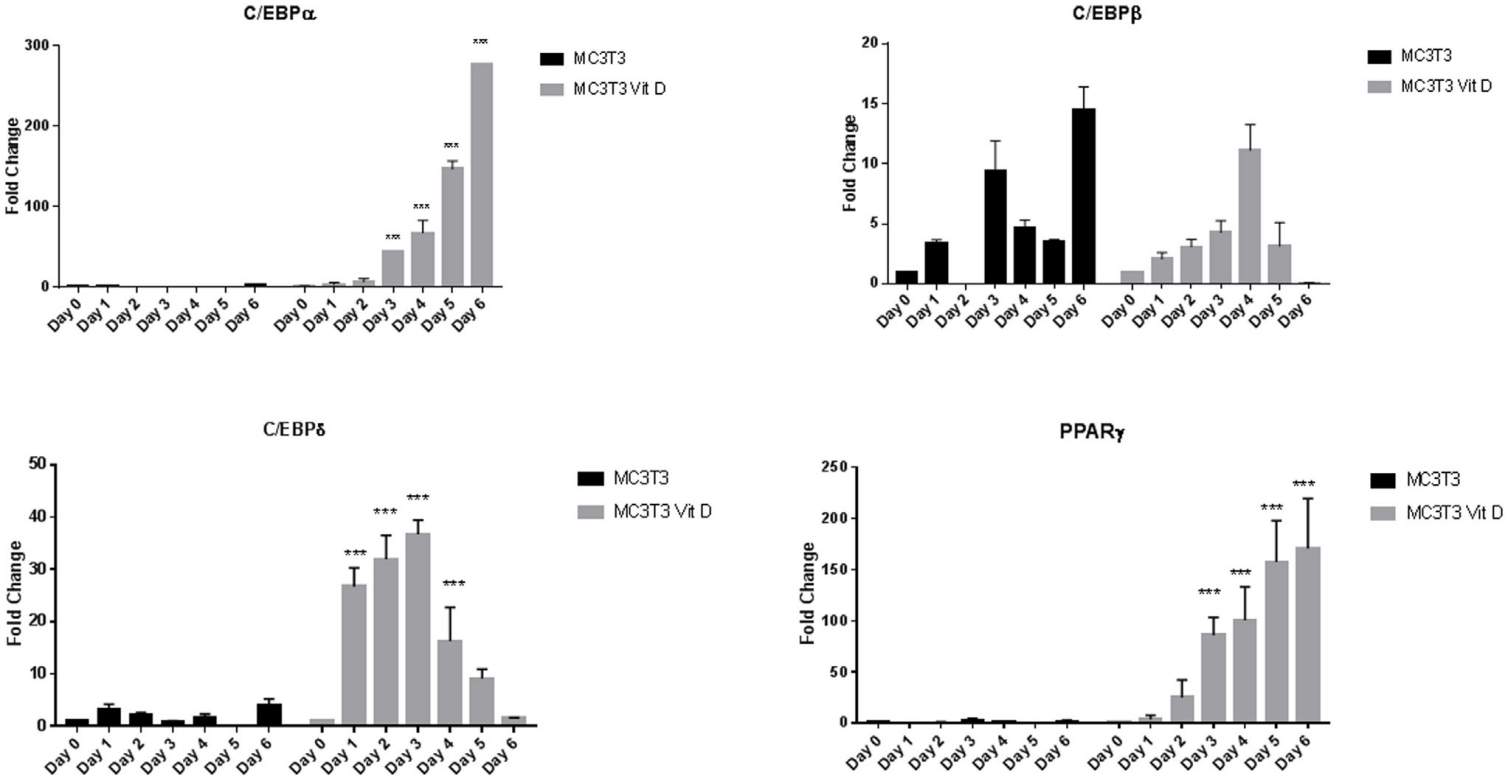
Real-time PCR of MC3T3-E1 cells exposed to adipogenesis-promoting media with and without vitamin D. Details of the experiment were similar to that described under Figure 1. Vitamin D was used at a concentration 20 nM with regular changes in media. Statistical significance is depicted in the body of each panel. Vitamin D-treated groups statistically significant from control untreated groups (***P < 0.001).

To determine the effect of vitamin D on osteoblasts in the presence of adipogenic differentiation factors, we exposed cells to 20 nM vitamin D for a week with media change every other day. Real-time PCR analyses of adipocyte transcription factors revealed a large upregulation of expression with vitamin D treatment. Figure 2 shows changes seen with and without vitamin D treatment of MC3T3-E1 cells. The overall sequence of expression of the transcription factors followed what we saw in cells without vitamin D. However, there was a dramatic statistically significant (*P* < 0.001) interaction between media and vitamin D in the upregulation of several factors. The only transcription factor that did not appear to be affected by vitamin D was C/EBPβ, which showed a change similar to that of cells treated only with adipogenic media. C/EBPδ showed about a 50-fold increase, PPARγ a 200-fold increase, and C/EBPα a 300-fold increase in activity when compared to cells treated only with adipogenic media.

**Figure 3:**
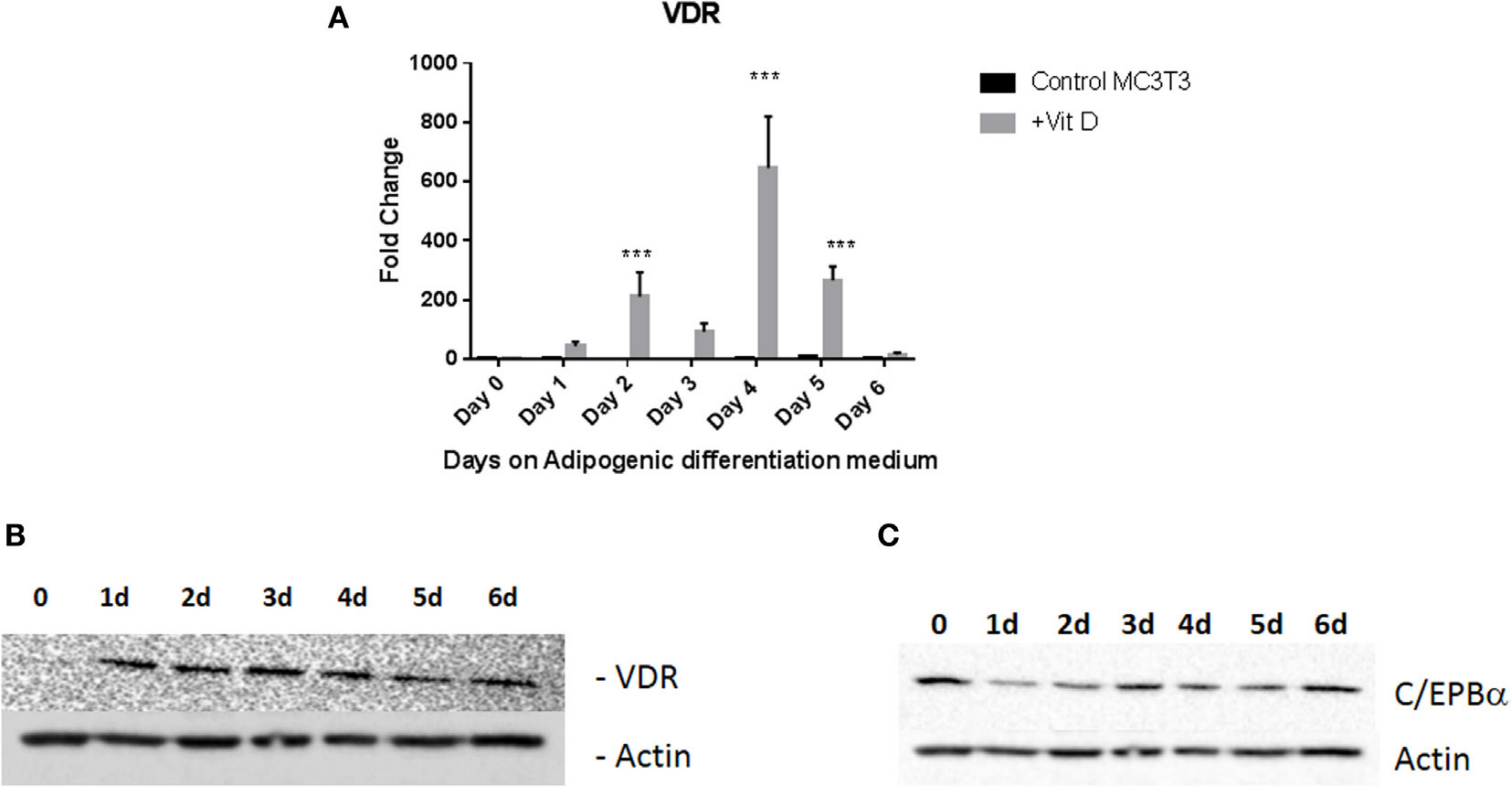
Vitamin D receptor (VDR) expression levels during adipogenic differentiation. **(A)** Real-time PCR of VDR mRNA expression after treatment with vitamin D. Cells were treated with 20 nM vitamin D or EtOH in adipogenic media and analyzed after regular changes in media VDR expression of control cells were compared to vitamin D-treated cells after 6 days. Vitamin D treatments that produced statistically significant increases in VDR expression are indicated ***P < 0.001. **(B)** Representative Western blot analyses of VDR levels after treatment with vitamin D. Treatment is similar to that described in Figure 2. Protein lysates from the treatment were analyzed using an antibody to VDR. β-Actin was used as a loading control. **(C)** Western blot analysis of C/EBPα after vitamin D treatment.

To confirm that this was a result of vitamin D receptor mediated action, we measured expression of vitamin D receptor (VDR) during vitamin D treatment. We treated MC3T3-E1 cells with adipogenic media with 20 nM 1,25 dihydroxyvitamin D with frequent changes of media as described in Section “Materials and Methods.” Control cells received the same amount of vehicle (EtOH). Addition of vitamin D produced a maximal increase of 600-fold 4 days (*P* < 0.001) after treatment with media with other days showing several fold elevation of expression (Figures 3A,B), demonstrating that the action of vitamin D did indeed result in an increase in its cognate receptor levels. Western blots for PPARγ (not shown) and C/EBPα (Figure 3C) also confirmed the changes seen in RNA expression.

### Expression of Adipogenic Marker Genes

**Figure 4:**
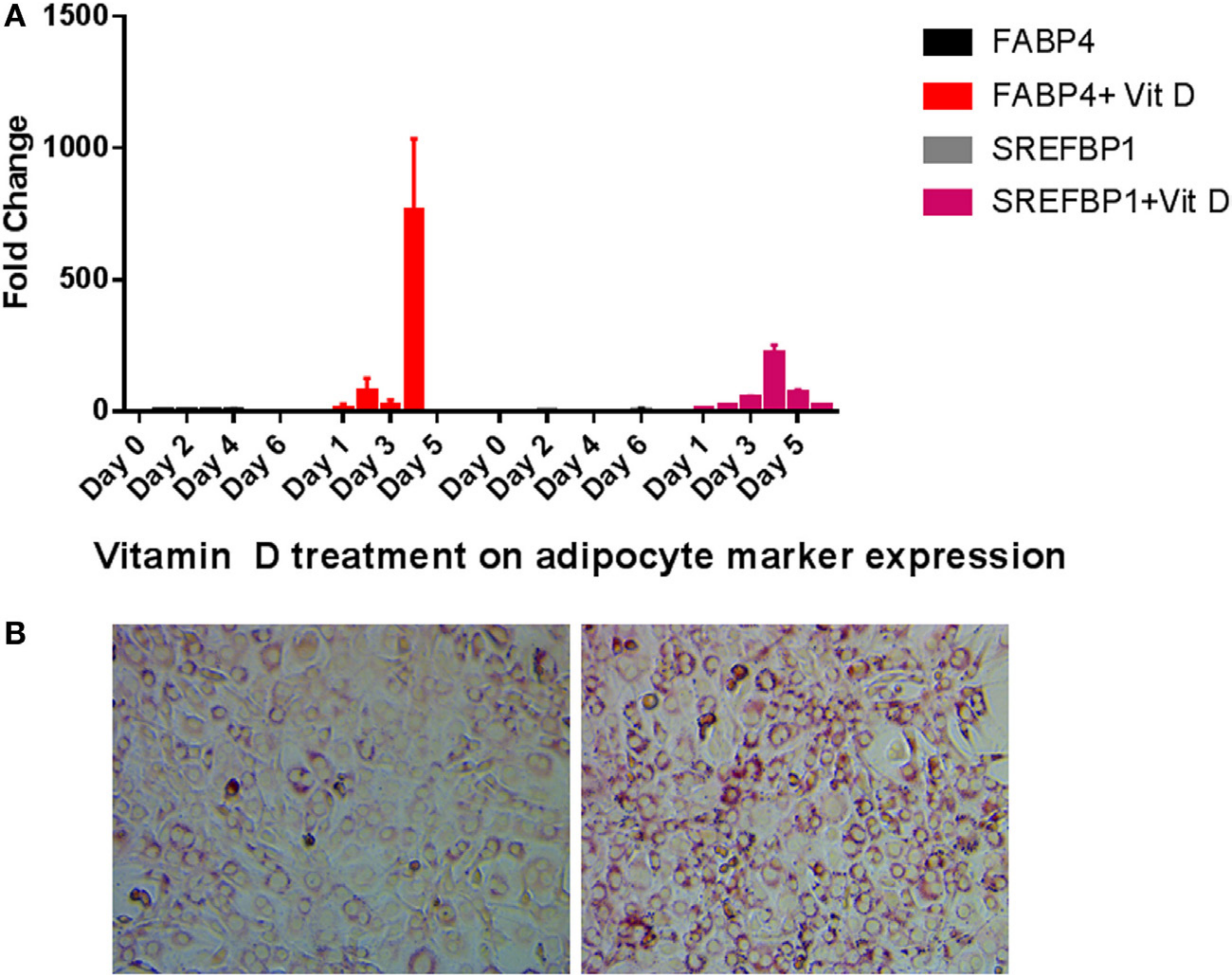
Adipocyte marker expression levels during vitamin D treatment in the presence of adipogenesis promoting (AP) media. **(A)** Real-time PCR of adipocyte markers after vitamin D treatment. Other details are similar to that shown in Figure 2. All statistics were calculated relative to control with no vitamin D treatment with O time set at 1. **(B)** Oil Red O staining of cells after vitamin D exposure demonstrates increased fatty vacuoles (left). Control cells received AP media and vehicle with no vitamin D (right) 20×.

We tested the expression of FABP4 and SREFBP1 after vitamin D treatment. When compared to untreated cells, treatment with vitamin D produced dramatic increases in FABP4 and SREFBP1 expression (Figure 4A). Transcripts for both these markers were increased by vitamin D treatment in the range of 100-fold and higher. Consistent with these results, we observed an increase in lipid droplet formation when cells were grown in the presence of vitamin D. Figure 4B showing that vitamin D was able to stimulate the transdifferentiation of osteoblasts to adipocytes when cultured in the presence of adipogenic differentiation medium.

### Effect of pRb1 Deficiency on Adipocytic Differentiation of Osteoblasts

**Figure 5:**
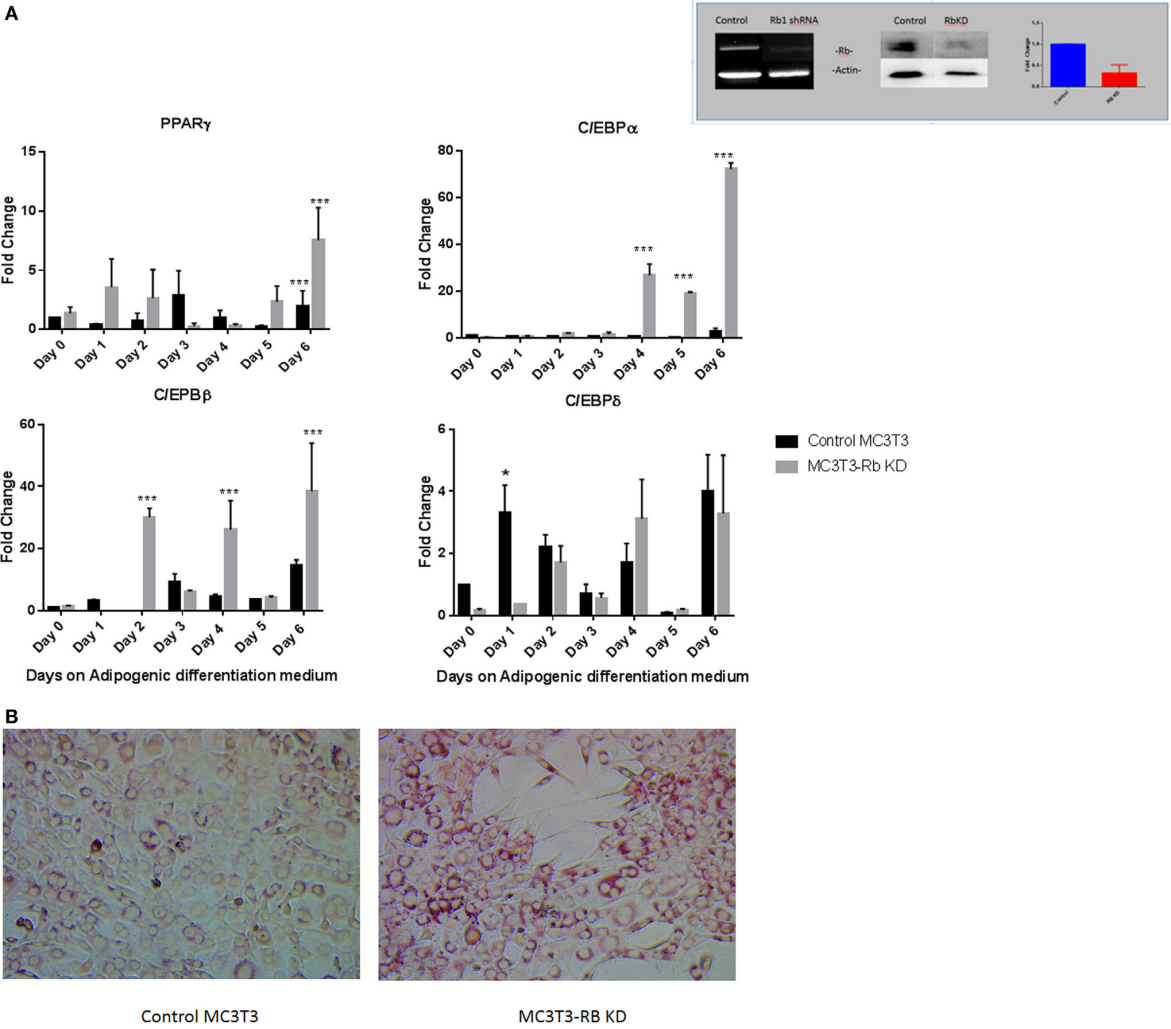

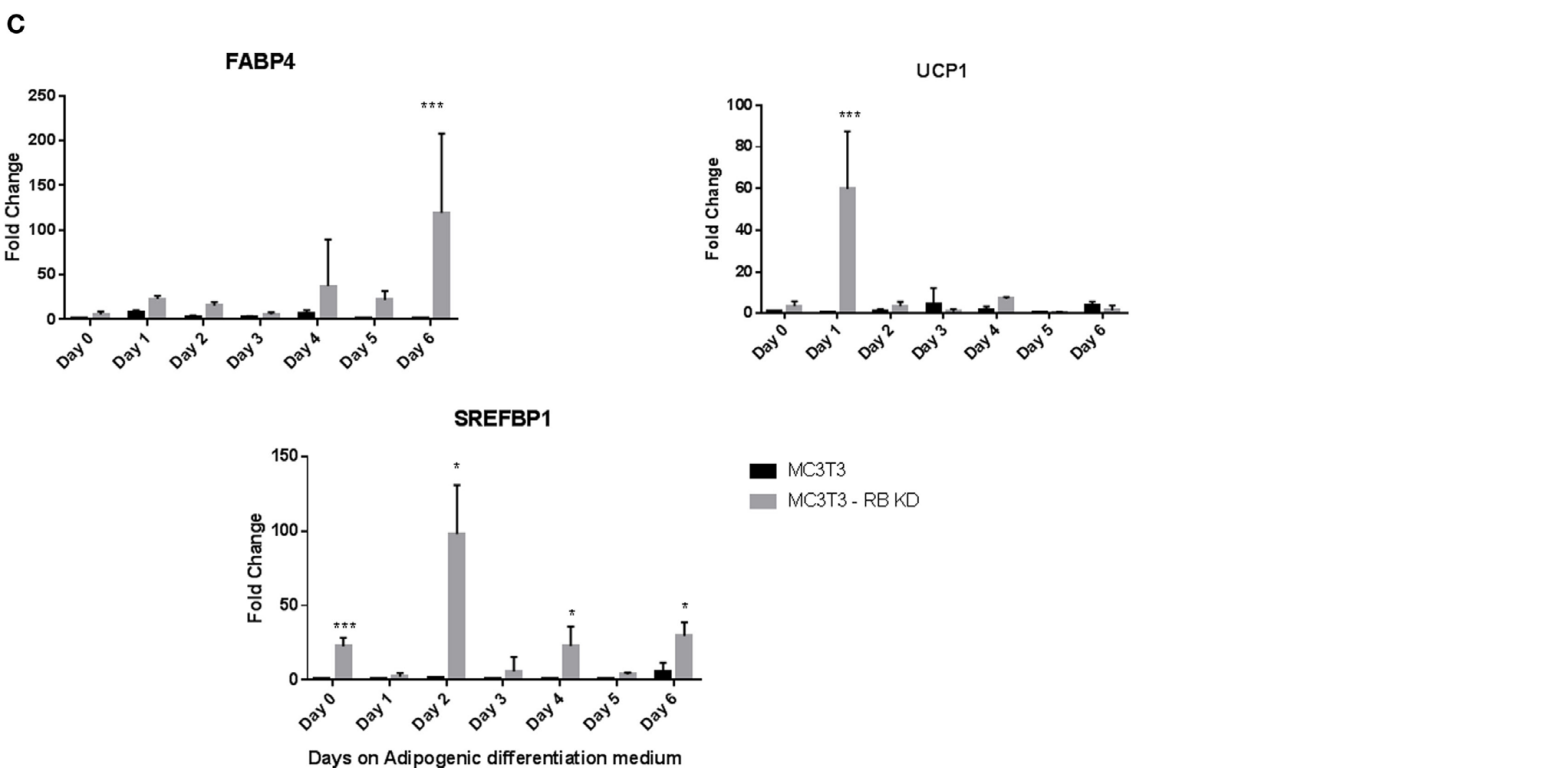
**(A)** Effect of Rb1 deficiency on adipocyte differentiation of MC3T3-E1 cells. Real-time qPCR of MC3T3 and MC3T3-RB KD of various adipogenic differentiation factors after the exposure to adipogenic media for various days. Results are expressed as mean ± SE (*n* = 3, *P < 0.05, and ***P < 0.001). The inset depicts qualitative and quantitative characterization of MC3T3-E1-Rb KD cells at the level of RNA and protein generated by shRNA technology. **(B)** Oil Red O staining of MC3T3-E1 and MC3T3-RB KD cells after exposed to adipogenic differentiation medium 20×. **(C)** Real-time PCR of adipogenic markers in Rb KD cells. Other details are similar to that in Figure 1. Results are expressed as mean ± SE (*n* = 3, *P < 0.05, and ***P < 0.001).

While our results with vitamin D suggested that the manipulation of preosteoblast MC3T3-E1 cells to express an adipocytic program is influenced dramatically at the level of transcription even though adipogenic genes are not known to be under hormone regulation by vitamin D. Therefore, we sought to determine if perturbation of key genes known to regulate adipogenesis might also produce a similar outcome. Recent studies have suggested that the presence of Rb1 is essential for osteoblast cell fate (14, 15). Other studies have demonstrated a role for pRb in the regulation of C/EBPs (13). pRb is also known to suppress PPARγ activity and activate Runx2 a osteoblast master regulator during mesenchymal cell differentiation (14). In this study, we attempted to determine how exposure to adipogenic media might affect osteoblasts with a deficiency in Rb1 expression. Figure 5 inset shows the generation and validation of Rb knockdown in MC3T3-E1. Other than a slight increase in growth rate (1.18-fold higher than control cells receiving scrambled control) that was not statistically significant (not shown), the cells did not appear to have any discernable changes to morphology. Several clones were analyzed and characterized, and we used one such clone to determine the effect of Rb deficiency on adipogenic program. As shown in Figure 5A, PPARγ expression trended toward being generally higher in Rb KD cells when compared to control cells but the data appeared highly variable. C/EBPα and C/EBPβ were also generally higher in Rb1 deficient cells and showed a clear increase with Rb loss (*P* < 0.001) during exposure to adipogenic media. On the other hand C/EBPδ appeared to be unaffected by Rb1 loss (Figure 5A). The increase in adipocyte differentiation of the Rb1 knockdown cells was also confirmed with Oil Red O staining (Figure 5B) and the expression of adipocyte makers FABP4, UCP1, and SREFBP1 (Figure 5C). We included UCP1 a marker of brown adipose tissue in our analyses as pRb has been shown to play a role in white and brown adipogenesis (13). While the extent of elevation of these markers was moderate when compared to what we observed with vitamin D treatment, nevertheless deficiency of Rb1 expression produced 20-fold to 100-fold increase in markers such as FAB4, UCP1, and SREFBP1 (Figure 5C).

**Figure 6:**
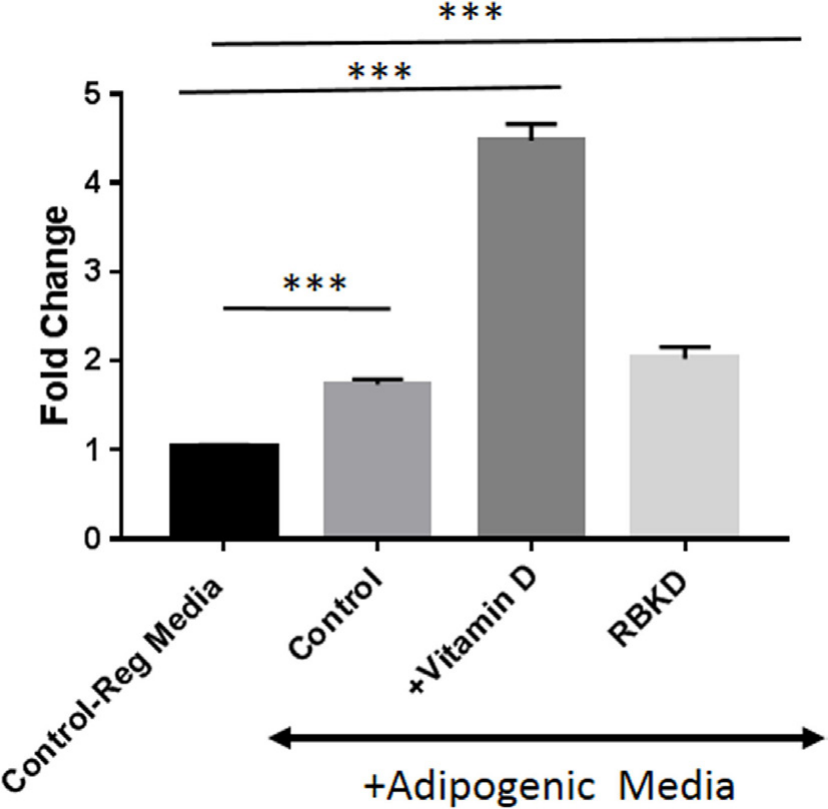
Peroxisome proliferator-activated receptor-gamma (PPARγ) promoter luciferase activity: cells were transfected with the PPARγ luciferase construct, and 72 h later luciferase activity was measured after incubation with and without vitamin D in the presence of adipogenesis promoting (AP) media. One set of control cells received regular media (10% fetal bovine serum + DMEM) and was used to compare the effect of either adipogenic media alone [control on AP media, or with vitamin D (+vitamin D) in the presence of AP media]. Rb KD cells were also exposed to AP media to study the effect of Rb1 loss on PPARγ activity. Results are expressed as fold change compared to activity in regular media and represent mean ± SE (*n* = 3, ***P < 0.001 comparing treatments vs. control regular media).

To confirm that these manipulations actually produced changes in PPARγ function, we conducted a luciferase assay with cells used in the above experiments. A construct containing six copies of PPARγ response elements were transiently transfected into cells followed by exposure to adipogenic media for 3 days in the presence and absence of vitamin D. As shown in Figure 6, maximal activity was seen after vitamin D treatment but just exposure to AP media was sufficient to produce a statistically significant (*P* < 0.001) increase in activity in control MC3T3-E1 cells. Loss of Rb1 expression also resulted in a statistically significant increase in PPARγ activity (*P* < 0.001) (Figure 6).

## Discussion

Adipocytes and osteoblasts share a common precursor in the bone marrow, and it is known that there is a high degree of plasticity between the two cell lineages (1). MC3T3-E1 is a clonal preosteoblast cell line that was originally isolated from new born mouse calvaria (17). To demonstrate plasticity in this line, investigators have generally employed genetic manipulations that have included overexpression of adipogenic transcription factors (18), treatment with drugs (19), etc. In this study, we attempted to understand the influence of adipogenic media with and without vitamin D or the effect of reduction in Rb1 expression on MC3T3-E1cells. Our studies show that exposure of these cells to media generally utilized to promote adipogenesis in preadipocyte cell lines such as 3T3-L1 can produce gene expression characteristic of adipocytes in determined preosteoblasts. However, we were surprised to find a synergistic enhancement of adipocyte differentiation factors and associated markers on treatment with vitamin D. While the role of vitamin D receptor and vitamin D on adipogenesis has not been clearly elucidated, it is well known that vitamin D receptors are necessary and present in adipocytes (20, 21). In fact, studies have demonstrated an inhibition by vitamin D of adipocyte specific expression of PPARγ and other factors in preadipocytes (22). None of the other transcription factors involved in adipocyte differentiation are also known to be regulated by vitamin D. Since VDR is expressed in response to vitamin D treatment, it appears that some of the effect could be mediated at the level of ligand receptor interaction. However, it is also known that vitamin D might have non-genomic actions (23) that may not involve the classical steroid hormone mediated stimulation of gene expression. Irrespective of the mechanism, treatment of preosteoblasts with vitamin D did not inhibit but promoted adipocyte differentiation. We cannot exclude the fact that adipogenic media components 3-isobutyl-1-methylxanthine and insulin may have played a role in influencing the adipocytic phenotype of these cells (20, 21).

While the final outcome of MC3T3-E1 cells exposed to adipogenic media was the same with and without vitamin D, there were important differences in expression of C/EBP factors. In the case exposure to just the media, the changes in gene expression agreed with previously published observations during preadipocyte differentiation to adipocytes (24). With vitamin D, our results show a dramatic increase in the case of C/EBPα, which went up several hundred fold and C/EBPδ which was increased 20-fold to 40-fold over just the media alone. While the extent of changes seen in C/EBPβ after exposure to AP media was the highest, with addition of vitamin the effect remained the same without any enhancement we saw in CEBPα and C/EBPδ (Figure 2). In a preadipocyte cell line 3T3-L1, liganded vitamin D appears to inhibit CEBPβ while unliganded VDR is seen necessary for adipogenesis (21). Our results may represent an inhibition of CEBPβ expression by vitamin D treatment, but the fact that upregulation provided by the media alone was still retained, might explain the overall enhancement in adipogenesis.

Previous work has suggested that the tumor suppressor gene Rb1 might be critical for cell fate determination and it has been shown that loss of pRb1 in mesenchymal stem cells cause them to default to an adipocytic cell lineage (14). We therefore used a cell line that has stable reduction in Rb1 expression to demonstrate that a similar phenotype of enhanced adipogenesis was seen. Interestingly, in these cells, there was a steady state increase in PPARγ expression and agrees with previous work that suggests that pRb inhibits PPARγ expression in mesenchymal stem cells (25). C/EBPα and C/EBPβ expression was generally increased with Rb1 loss, but C/EBPδ expression was unaffected. This was unlike our observations with vitamin D and might relate to independent roles of Rb1 in regulating these transcription factors. It is also known the Rb1 regulation of adipogenesis might depend on several factors, since pRb regulates not only the CEBPs but also cell cycle control through E2Fs. In addition, loss of Rb1 promotes brown adipose tissue conversion as evidenced by markers such as UCP1 by these cells (Figure 5C).

Our studies generally reveal that uninduced preosteoblasts maintain their plasticity toward adipocytes and factors in the adipogenic media appear to be sufficient to induce MC3T3-E1 cells to express the adipocyte phenotype. While this is not surprising, what is surprising is the fact that treatment with vitamin D in the presence of adipogenic factors dramatically enhances gene expression in preosteoblasts toward adipocytes. While this result was unexpected, studies that have looked at genomic binding of transcription factors in differentiated and undifferentiated cells demonstrate that this might relate to the plasticity of the epigenome (26). Both histone modifications and transcription factor binding appear to be different in mature versus immature osteoblasts (26, 27). While there may be a default pathway or preference for a particular cell fate, it appears to be easily perturbed at the level of transcription factor binding (26). In fact, these investigators show that transdifferentiation of preosteoblasts to adipocytes is associated with rapid changes to chromatin in regions containing the adipocyte specific genes in MC3T3-E1 cells. It is also known that unliganded VDR remains bound to a number of target genes with the genome and undergo modifications with vitamin D treatment. It is possible that vitamin D treatment might not have directly contributed to the activation of adipocyte specific gene expression but may have altered the transcription factor binding sites of VDR and essentially mitigated the inhibition of VDR targets. It is known that vitamin D exerts both positive and negative effects on osteoblast differentiation (25) and is dependent on timing. In immature osteoblasts, vitamin D downregulated the master regulator of osteoblast differentiation Runx2 while increasing PPAR activity and adipocyte differentiation (28). While our studies have not investigated in parallel how osteoblast differentiation may be affected in the presence of adipogenic media and vitamin D, it is likely that competition of adipogenic and osteogenic transcription factors for binding on DNA might have influenced the response seen. There is much to be learned about vitamin D targets as several of the sites where VDR bound on chromatin is not known targets of vitamin D action (27). Our results suggest the need for further functional studies to uncover vitamin D-dependent pathways that govern adipocyte–osteoblast transdifferentiation.

## Author Contributions

EP designed and performed experiments, analyzed data, and helped write the paper; NC designed experiments, analyzed data, and wrote the paper.

## Conflict of Interest Statement

The authors declare that the research was conducted in the absence of any commercial or financial relationships that could be construed as a potential conflict of interest.
